# Multi criteria analysis of municipal solid waste management and resource recovery in Poland compared to other EU countries

**DOI:** 10.1038/s41598-023-48026-3

**Published:** 2023-12-12

**Authors:** Viola Vambol, Alina Kowalczyk-Juśko, Sergij Vambol, Nadeem A. Khan, Andrzej Mazur, Marianna Goroneskul, Oleg Kruzhilko

**Affiliations:** 1 Department of Environmental Engineering and Geodesy, University of Life Sciences in Lublin, Lublin, Poland; 2 Department of Applied Ecology and Nature Management, National University «Yuri Kondratyuk Poltava Polytechnic», Poltava, Ukraine; 3 Department of Occupational and Environmental Safety, National Technical University Kharkiv Polytechnic Institute, Kharkiv, Ukraine; 4 Interdisciplinary Research Center for Membranes and Water Security, King Fahd University of Petroleum and Minerals, Dhahran, 31261 Saudi Arabia; 5 Department of Physical and Mathematical Sciences, National University of Civil Defense of Ukraine, Kharkiv, Ukraine; 6 Department of Ecology and Economics of the Natural Environment, Technical University “Metinvest Polytechnic” LLC, Zaporizhzhia, Ukraine

**Keywords:** Energy and society, Environmental impact

## Abstract

Statistics show that the inhabitants of Poland are producing increasingly more household waste. This article attempts to determine the current level of development of Poland in the field of waste management concerning other EU countries and partner countries; identify trends in the mass of generated, segregated, and mixed municipal waste; and obtain an idea of the attitude of the Polish population toward sorting waste at the source to bring the country to a higher level of waste management. The empirical base is statistical data published on the website of the EU Data Explorer and the Central Statistical Office. The ranking of countries was determined by the TOPSIS method using a synthetic indicator based on the selected diagnostic features. The significance of the obtained ranks was tested using the non-parametric Friedman test (p < 0.01). We established that Poland has been consistently ranked 16th-17th over the past 5 years. Unfortunately, thus far, no systematic approach has been found to raise citizens' awareness, which may be due to the lack of the necessary amount of data. Researchers recommend investigating the sensitivity of the relationship between the generation of alternative energy from waste and the authorities' action.

After the expiration date or service life, each product a resident purchases turns into waste. Such waste is classified as municipal solid waste. Numerous studies in different countries^1–9^ show that most household wastes comprise biodegradable components, which, when buried, contribute to soil, surface, and groundwater pollution and greenhouse gas emissions. Reducing adverse effects is possible by preventing waste generation and rationally managing it, a vital component of the circular economy (CE)^10^ and is in line with the European Green Deal^11,12^. Following Directive 2008/98/EC^13^, waste disposal in landfills is possible only if it is justified that these wastes are unsuitable for recycling or any other disposal, and this management method provides the best environmental result.

Waste management is a critical element of the bioeconomy, allowing the natural environment to be kept clean, combined with the use of recycled materials, thus bringing economic benefits. Countries' transition to a circular economy should reduce the pressure on natural resources and create sustainable growth and jobs. It is also necessary for achieving the EU goal of climate neutrality by 2050 and halting biodiversity loss^14^. The new action plan announces initiatives throughout the product lifecycle. This plan focuses on how people develop the products, promote circular economic processes, encourage sustainable consumption, and aim to prevent waste and preserve used resources in the EU economy for as long as possible. The plan introduces legislative and nonlegislative measures targeting areas where action at the EU level is of real benefit.

The European Commission advocates for the EU to follow an approach that prioritizes waste prevention. The principles of reuse, recycling and energy recovery are central and complementary, and landfilling should be avoided^15^. Following the green track, waste generation trends in Europe are relatively stable, but societies still produce significant amounts of waste. Although the waste rates differ significantly between different EU countries and partner countries, at the same time, the share of waste sent to landfills is decreasing, while the amount of waste for recycling is steadily increasing^16^. According to 2017 statistics, 46% of all household waste in EU countries and partner countries is recycled or composted, but waste management practices vary widely across EU countries and partner countries; several countries still bury large amounts of household waste in landfills^17^. Statistics show that the inhabitants of Poland are producing increasingly more household waste^18^. At the same time, at least 91% of the country's electricity is produced at thermal power plants running on fossil fuels such as coal, hydrocarbons, and brown coal. Consequently, Poland’s transition to renewable energy sources, in particular from waste, is urgently needed. So, the decision on which CE-related strategies, policies and technologies to adopt is critical as it can change the dynamic interactions between all economic actors, including city waste management networks^15^.

Since household waste management is a multi-criteria phenomenon, covering various types of activities to reduce the amount of waste, the effectiveness of such management of household waste can be characterized using various simple signs (waste management operations influencing their quantity) and, on this basis, assessed using synthetic indicators (measures). Therefore, it is necessary to carry out a multi-criteria analysis to understand Poland's development level in waste management and the issues of waste management as a potential energy source.

At present, many studies have already been carried out to study the indicators of the mass of waste in Poland^19^, the level of development of individual voivodeships in the field of waste management^20^, the study of the morphological composition of waste in Poland^21^, the study of the state of knowledge, the level of awareness and attitudes of Polish residents to the economy through a survey^22^, and even examining factors that determine consumer participation in the fight against food waste in restaurants in Poland^23^. However, all these studies were carried out for a specific short period; as a rule, they had a geographical limitation and did not provide an opportunity to see the general situation of Poland's development in this matter. That is, no research has been carried out so far that would show the systemic advantages and disadvantages of the current approaches taken in Poland to manage waste as a potential source of energy to follow the EU strategic plans. At the same time, the study's authors found a positive impact on the economy by increasing the efficiency of resource use, which can ultimately can reduce energy dependence and improve the population's quality of life^24^.

Thus, the article attempts to determine Poland's current level of development in manage waste as a sustainable resource for energy production concerning other EU countries and partner countries and the trend in the mass of generated segregated and mixed municipal waste. The first purpose is to conduct a study by quantitative methods using bibliometric analysis to understand the topic relevance and identify relationships using keywords, followed by the second purpose: based on the multi-criteria decision making method (MCDM), to determine Poland’s place in the ranking among other European countries and partner countries and to mathematically substantiate the significance of the ranking results. Comparison of the rating results with the Polish policy pursued in the studied time interval will allow us to identify effective approaches to waste management and adjust these approaches for the development of the waste management field. Accordingly, the study aims to answer the following questions:

- *Question 1*: Can municipal solid waste be a sustainable source of energy in Poland?
- *Question 2*: What is the level of development of Poland in the field of solid waste management in comparison with other European countries and partner countries, since all these countries follow the same green economy strategies and course, and what are the prospects for the future?
- *Question 3*: What is the attitude of the Polish population towards waste segregation at source of generation, as it is one of the effective waste management approaches that promote the development of waste-to-energy to take the country to a higher level in this field.

## Literature review

### Data collection procedure

This section contains information about the data collection procedure that was applied for the bibliometric analysis. Data were collected according to the protocol presented in Table 1.

**Table 1:** Data collection.

Databas	Scopus (Elsevier)
Keywords (request formula)	"Municipal solid waste" AND ( poland OR ec OR "European Union" )
Search field	Title, abstract, keywords
Publication type	All
Publication language	All
Time interval	2014–2021

This analysis was conducted on documents indexed in Scopus (Elsevier), an accessible and widely distributed database of peer-reviewed scientific publications. A search conducted only in Scopus is considered valid, despite the fact that Elsevier includes two different databases, Scopus and ScienceDirect. This is justified by the fact that Scopus indexes in almost the entire ScienceDirect database (https://service.elsevier.com/app/answers/detail/a_id/28240/supporthub/dataasaservice/p/17729/).

Combination with publications from Web of Science was not carried out in order to avoid unnecessary repetitions, since the coverage of Scopus is greater than that of the Web of Science^25^ and in most cases, peer-reviewed scientific journals are registered in both databases.

The choice of keywords and query formula is justified by the fact that we were interested in current issues related to waste management in particular in Poland and the EU as a whole (to understand Poland’s place in the ranking of EU countries).

When determining the time interval under study, the authors were guided by the current situation in the world and existing pressing problems in the area under study. The choice of this time interval was influenced by three main factors:

1. in mid-2013, changes occurred in the waste management system in Poland, namely, a waste segregation system was introduced, and responsibility for waste management was transferred to municipalities. Thus, the first full year of reporting in the new waste management system was 2014;
2. since 2014, the problem of obtaining energy from waste is becoming increasingly urgent every year due to the emergence of an energy crisis due to the imposition of sanctions against Russia in connection with the annexation of Crimea. In previous years, Europe was dependent on Russian energy imports and the EU relied on energy imports from the Russian Federation^26^. Due to the imposition of sanctions and restrictions on the export of equipment for oil and gas production, the need to find our own resources has sharply increased in order to significantly reduce and subsequently eliminate this dependence;
3. the end date of the research time interval is justified by the lack of data for 2022 in the Eurostat database.

The search resulted in a list of 430 documents. Based on these obtained results, all categories of documents were evaluated. Among these documents, 417 were published in English, 7 in Chinese, 3 in Polish, 2 in Portuguese and 1 in German. Documents not published in English were also taken into account as they had an abstract and keywords in English.

Not all identified documents were devoted to the problems of solid waste management in Poland or the European Union countries and partner countries.

### Expert opinion process

Before proceeding with the biblometric analysis of the found documents, in order to reduce the risk of systematic error in this study, an expert review of the documents was carried out to ensure that the topics of these studies corresponded to the questions posed in the current study. Expert assessment, which is based not on opinions, but on information, is useful^27^. Six authors of the current study served as experts. All experts worked independently to review all retrieved documents and reported to the corresponding author of the article.

Based on the results of expert assessments, 81 documents were rejected as inappropriate and 349 documents were accepted for bibliometric analysis.

### Bibliometric analysis

Bibliometric analysis has become increasingly popular for research applications in recent years because it can identify new trends in research activity around the world, as well as explore the body of scientific knowledge and the evolutionary nuances of a scientific problem under study by accurately understanding large volumes of unstructured data. Many studies using bibliometric analysis demonstrate that these studies provide a solid basis for the development of a particular field by processing large volumes of scientific data and contribute to the achievement of high research impact^28^. Also, when conducting bibliometric analysis, the risk of bias is significantly reduced, since this analysis is based on quantitative methods.

**Figure 1:**
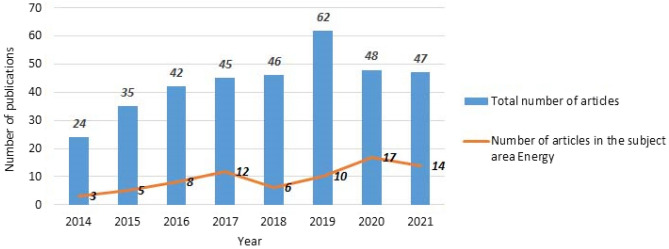
Distribution of scientific research on solid waste management issues published from 2014 to 2021, by year of publication (349 documents).

**Figure 2:**
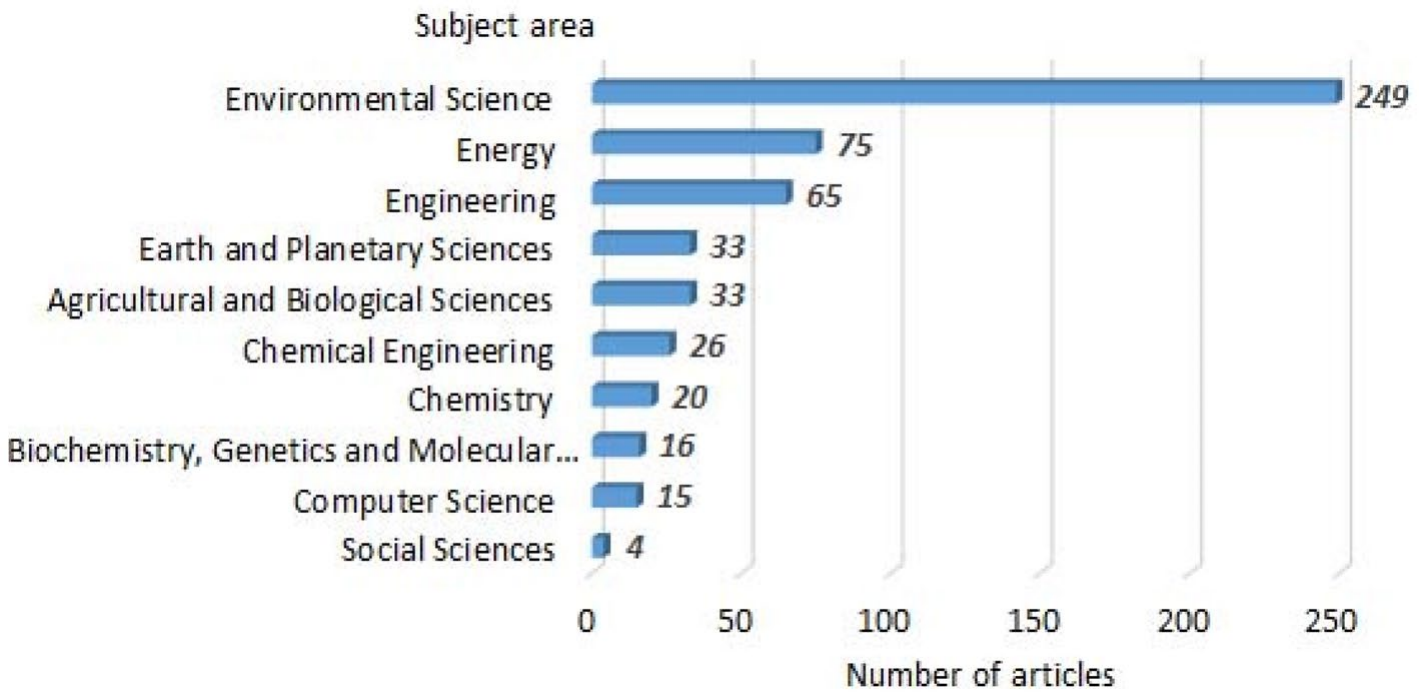
Distribution of scientific research on municipal solid waste management, published from 2014 to 2021, by subject area (349 documents).

For bibliometric analysis, the science mapping technique was used. The annual distribution of selected publications (from 2014 to 2021) is presented in Figs. 1 and 2 shows the priority areas of research related to waste management issues.

It is obvious that from 2014, academic interest in the problems of solid waste management increased until 2019, but in subsequent years the number of publications decreased. The peak of publications in the research area is observed in 2019—62 publications and the next year for the largest number of publications is 2020—48 publications.

Of these publications, sharp superiority is ensured in the subject area of Environmental Science—249 publications and the second highest priority subject area is Energy—75 publications, which indicates the relevance of these scientific problems.

**Figure 3:**
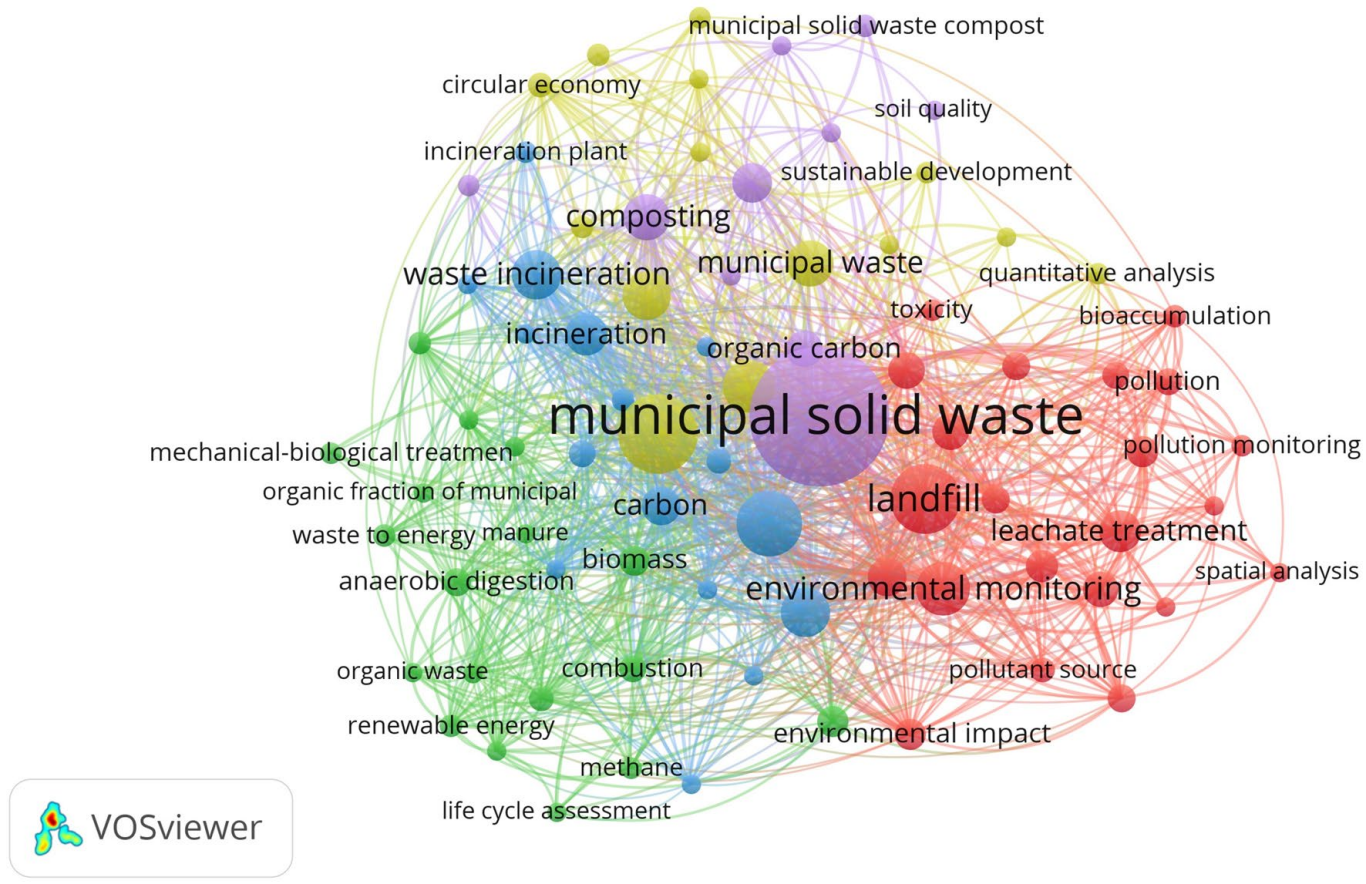
Analysis of matches by keywords of the created selection of documents (349 documents) for 2014–2021 related to solid waste management issues in Poland and EU countries.

Environmental Science is an interdisciplinary academic field that studies the physical, chemical and biological processes that occur on Earth, as well as the social, political and cultural processes that influence the state of the planet, and contributes to the search for solutions to environmental problems. Consequently, this subject area quite logically contains scientific research devoted to environmentally friendly management of household waste, including those related to its use as a resource for energy production. In this regard, a selection of documents was subjected to bibliographic analysis using the VOSviewer program to establish the most frequently occurring keywords and connections between them. The result of the analysis is presented in Fig. 3. Each node in the network represents a keyword, where the size of the node indicates the frequency of its occurrence. Relationships between keywords are shown. Thicker lines indicate stronger connections between keywords that appear most frequently in a pair. Terms within a cluster have stronger relationships with each other than with terms in other clusters.

**Figure 4:**
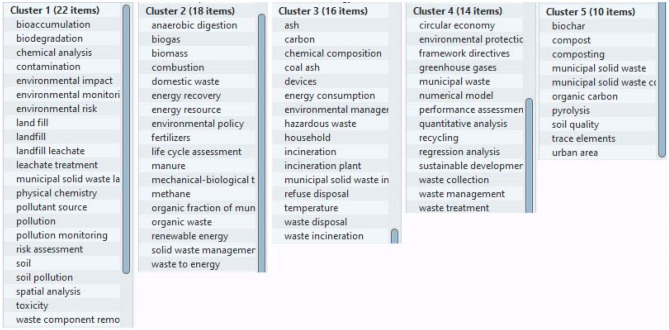
Identified keywords and connections between them that are most often repeated in the studied selection of publications.

The VosViewer application used identified 80 items (keywords), 5 clusters, 1272 links and a total link strength of 3454 (Fig. 4). Only relevant elements appearing more than 5 times in the selected 349 publications are presented. This analysis demonstrates that the most frequently occurring positions were “municipal solid waste”—190 cases, landfill (land fill, landfill leachate) with a total of 84 cases, followed by “waste management”—67 cases.

**Figure 5:**
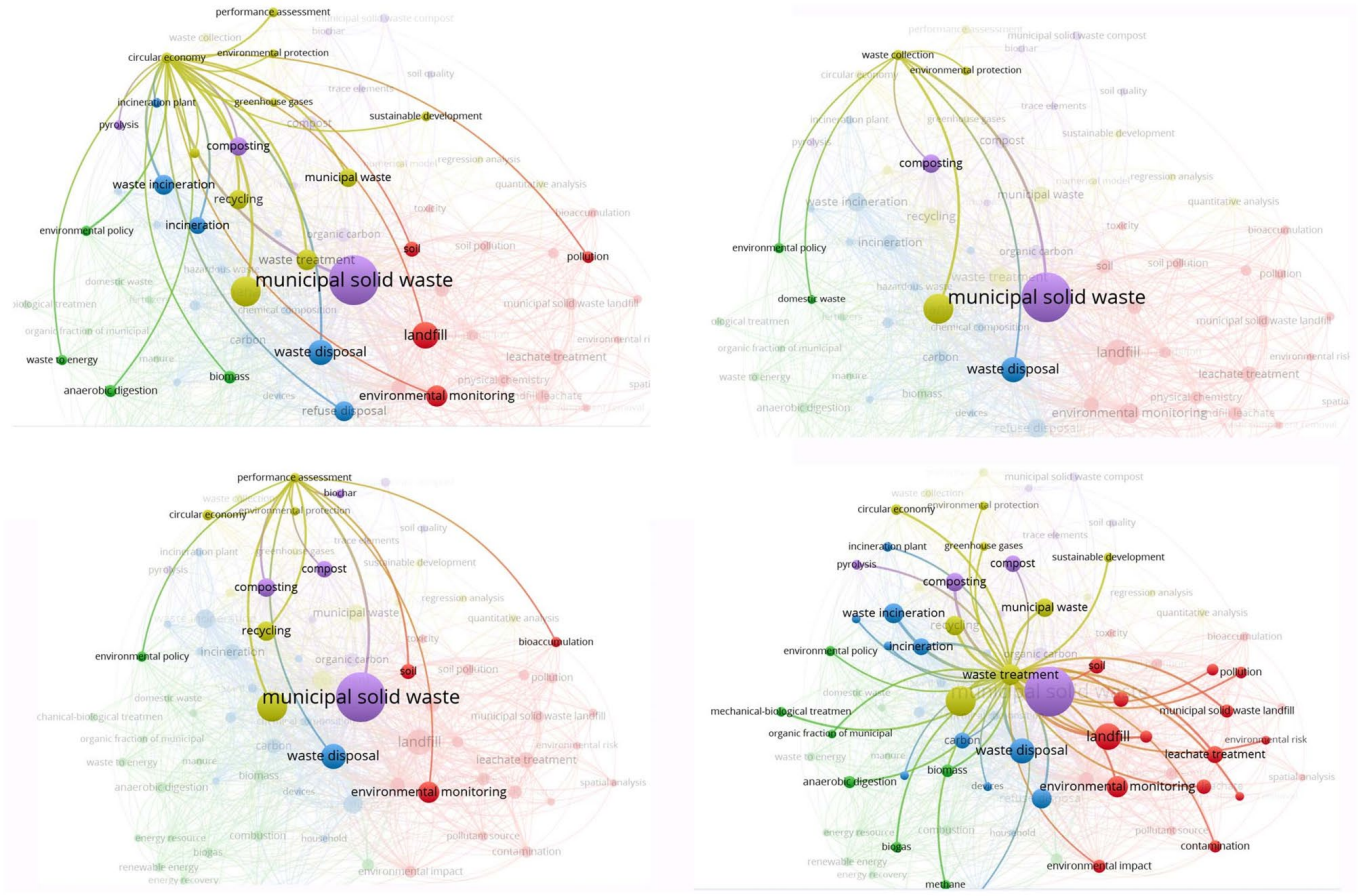
Yellow cluster of bibliometric analysis by keywords of a selection of documents (349 documents) from 2014 to 2021.

In Fig. 3, publications belonging to the yellow cluster can be considered as publications representing a framework dedicated to circular economy, efficiency assessment and the resulting waste collection and treatment methods (Fig. 5).

**Figure 6:**
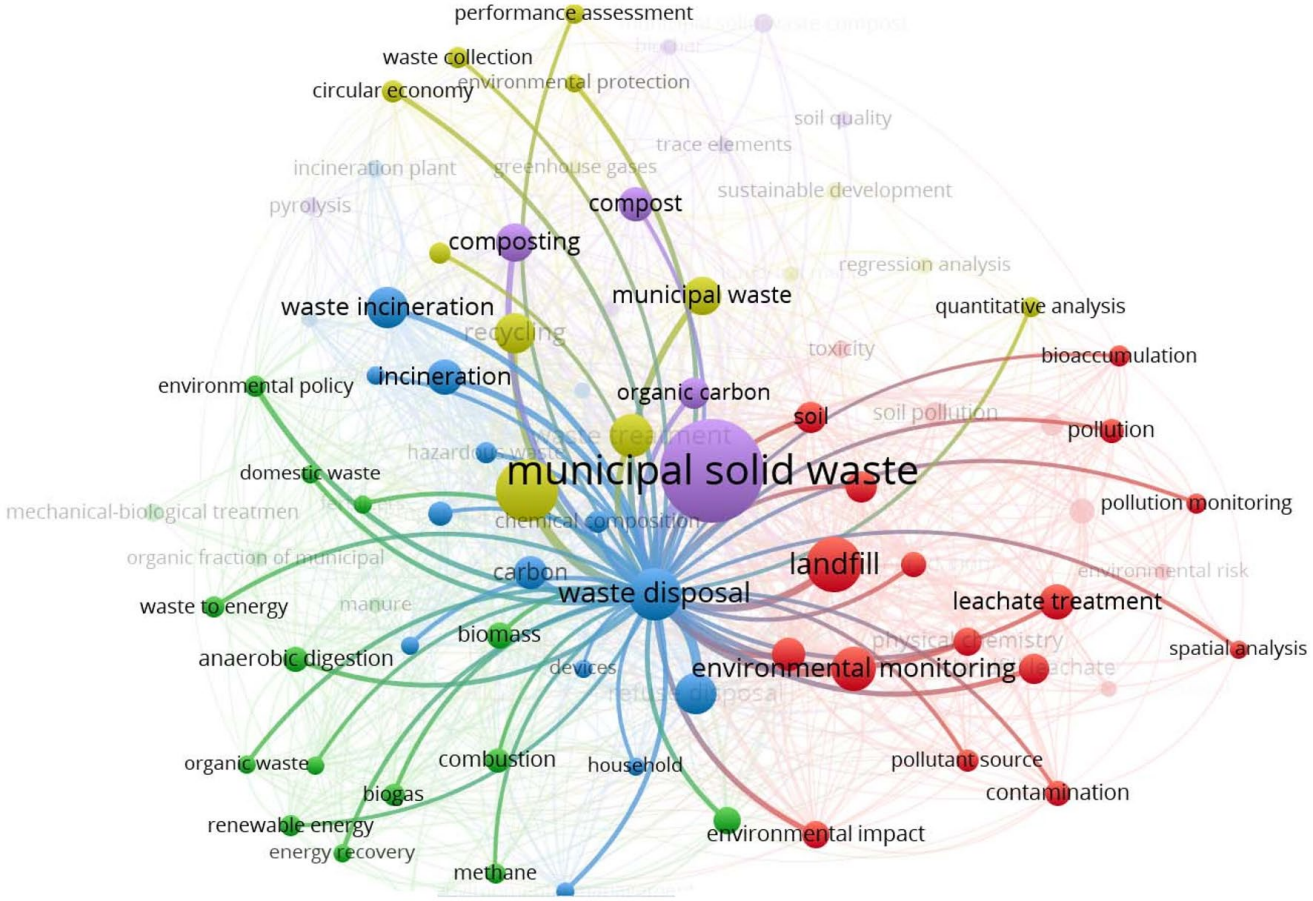
Blue cluster of bibliometric analysis by keywords of a selection of documents (349 documents) from 2014 to 2021.

The blue cluster contains what appears to indicate a model focused on waste treatment methods (including hazardous waste), primarily incineration and composting, as well as associated devices, temperatures and hazards (Fig. 6).

**Figure 7:**
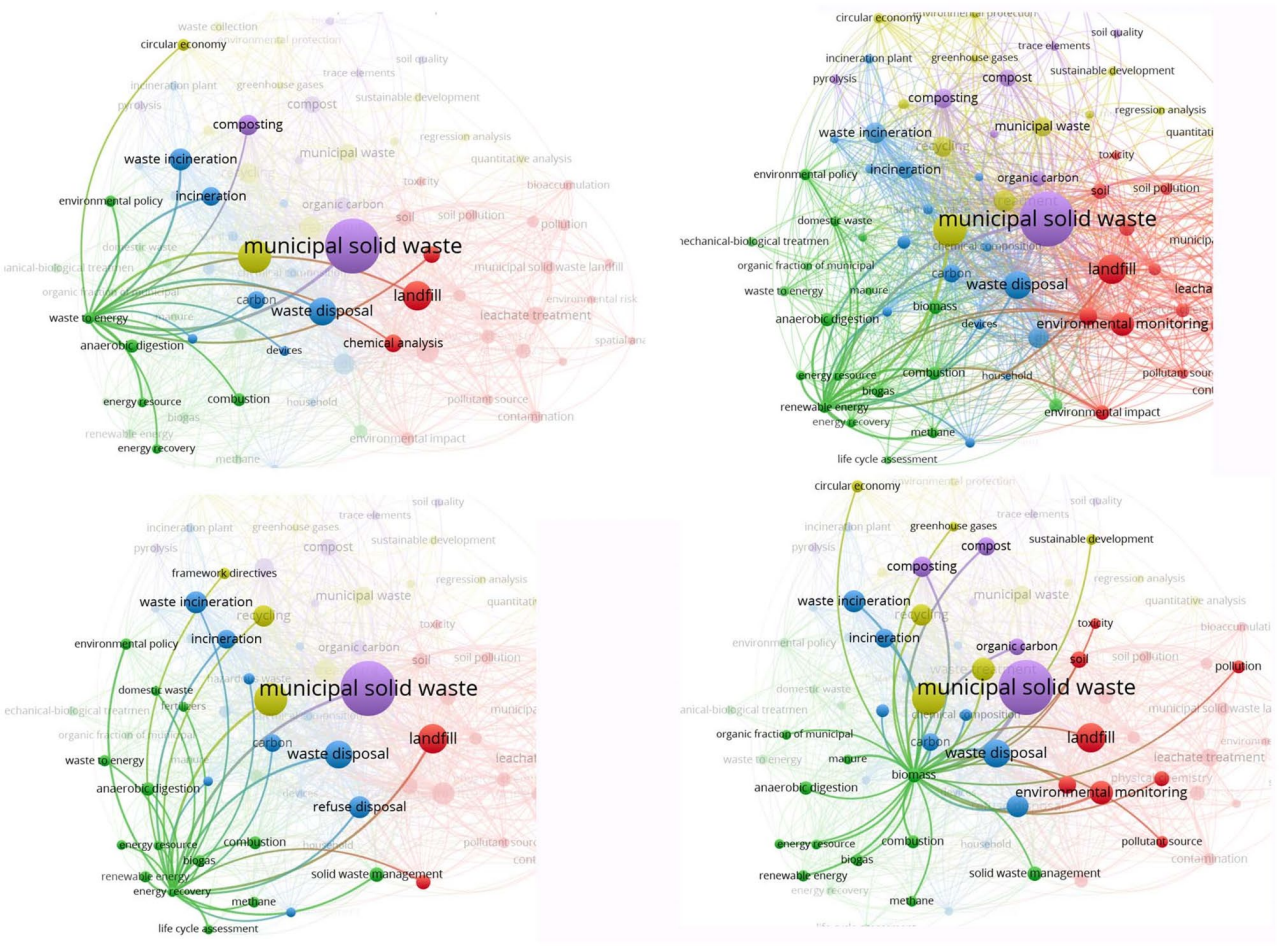
Green cluster of bibliometric analysis by keywords of a selection of documents (349 documents) from 2014 to 2021.

The red cluster is focused on environmental monitoring of the environment and waste management processes. The greatest number of references to municipal solid waste as a resource for energy production is found in the green cluster (Fig. 7). This cluster contains keywords such as anaerobic digestion—10 cases, biomass—11 cases, biogas—8 cases, combustion—9 cases, household waste 5 cases, carpet energy—5 cases, energy resource—5 cases, environmental policy—7 cases, life cycle assessment—5 cases, mechanical–biological treatment—6 cases, methane—6 cases, organic fraction of municipal solid waste (organic waste)—10 cases, revival energy—7 cases, waste to energy—7 cases.

**Figure 8:**
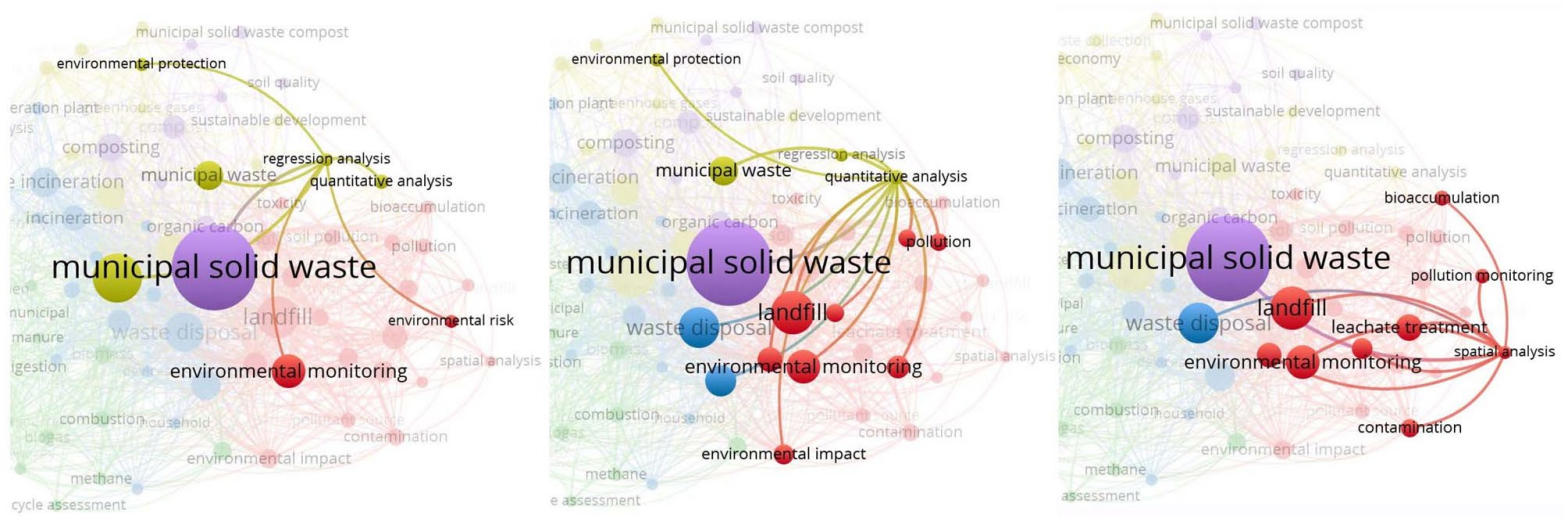
Methods of analysis identified using bibliometric analysis using keywords of a selection of documents (349 documents) from 2014 to 2021.

This analysis shows the relevance of scientific research in the field of converting solid household (or municipal) waste into energy. Also, you can see that in this selection of documents there are such research methods as regression analysis (5 cases) in relation to the assessment of waste generation and its treatment; quantitative analysis (6 cases) in relation to the assessment of pollution and environmental impact; and spatial analysis (5 cases) in relation to the assessment of waste accumulation, environmental monitoring and environmental pollution (Fig. 8).

However, there is a gap in research devoted to the critical assessment of approaches to waste management as a resource for energy production using the method of multi-criteria decision making, in other words, a multi-criteria assessment of the waste management system in the country.

The current study seeks to fill this gap.

## Materials and methods

### Data

In this study, the empirical basis is the statistical data on municipal waste by waste management operations, published on the EC data browser website^29^ by the Central Statistical Office (GUS)^30^. The current study used secondary data due to the difficulty of obtaining global data. This data comes from Eurostat, which reports waste management statistics for 38 countries (European Union countries and partner countries) based on 10 waste management operations from 1995 to 2021.

However, the research concerned 30 countries belonging to the European Union and partners (hereinafter referred to as EU countries). 8 countries were not included in the study due to lack of necessary data (justification is presented in Sect.  Research limits). Of the 10 waste management operations published on the EC data browser website, 4 were included in the study as diagnostic features (the rationale is presented in Sect.  Ranking of EU countries). The general preliminary analysis of the waste situation in Poland and in EU countries is limited to 2002–2021, and the time interval from 2014 to 2021 is used for comparative and trend analysis (justification is presented in Sect.  Data collection procedure).

### Ranking of EU countries

Multi-criteria decision making (MCDM) method provide an opportunity for a broad and objective view of various complex phenomena through their comprehensive study. MCDM is a structured approach to selecting the most suitable alternative, taking into account the importance of evaluation criteria and the performance of alternatives for each criterion. The different levels of importance of the criteria are described by assigning weights to them. One of these methods is TOPSIS—a method for determining the order of preference by similarity to the ideal solution.

The ranking of EU countries was carried from 2014 to 2021.

We constructed a synthetic indicator (measure) Q of the level of development of countries in the field of waste management as a sustainable resource for energy production using the multicriteria TOPSIS method, which Hellwig^31^ introduced to assess the phenomenon described by several indicators synthetically^31^. For the assessment, we identified diagnostic features that characterize individual countries in terms of the level of development in the field of waste management, separation of characteristics, normalization of feature values, calculation of the value of the synthetic measure of development, linear ordering of the unit and the definition of types of development. We carried out linear ordering based on interrank comparisons since this method is characterized by simple construction and high applicability in quantitative regional analyses^20^. This method is the basis for the preparation and interpretation of the ranking of EU countries in terms of the level of development in waste management as a sustainable resource for energy production in recent years.

As diagnostic features for ranking, we chose the values ${{\varvec{x}}}_{{\varvec{i}}{\varvec{j}}}$ (*i* = 1, …, *m* ; *j* = 1,…, *n*) of the average amount of waste per person (kg/capita) for waste management operations, which are presented in the statistical reports of Eurostat.

Eurostat reports statistics based on 10 waste management operations. Of all the operations, only those that have a complete representation in the database for a particular operation or country were taken into account. However, for some operations had significant data deficiencies. Thus, due to the lack of data in Eurostat on the required operations, 8 countries out of 38 represented by Eurostat were not taken into account when developing the rating. Also, the authors decided to exclude features that do not have a sufficient set of data and features that do not reflect the specifics of waste management (that is, those features that do carry general information without specifying the phenomena). Acceptance of specific diagnostic features was determined first by the amount of missing data for a particular operation or country, then based on the priority of the waste management operation within the current study (Table 2).

**Table 2:** Rationale for the selection of diagnostic features for the current study.

No	Waste management operations according to Eurostat data^29^	Description	Availability of data	Adoption of the operation as diagnostic features	Rationale
1	Waste generated	An indicator that demonstrates the total volume of waste generated	Yes	No	Does not reflect the specifics of waste management
2	Waste treatment	An indicator that demonstrates the total volume of waste processed using one of the methods listed below; in the statistical database the value of this indicator coincides with the value of the indicator above	Yes	No	Does not reflect the specifics of waste management
3	Disposal—incineration (D10) and recovery—energy recovery (R1)	These operations imply that the waste is used primarily as a fuel or other means of generating energy (D10) and (R1) by combustion with energy recovery	No	No	Lack of data for all countries and for the entire time interval
4	Disposal—landfill and other	Disposal of waste in landfills. The simplest and most common method of waste management, but the most unfriendly to the environment. Indicates irrational use of resources. As a rule, this method is actively used in underdeveloped countries	Yes	Yes	Demonstrates the level of development of the country quite well
5	Disposal—incineration	A widely used and uncomplicated method of waste management. It is often used to reduce the volume of waste accumulation in order to avoid excessive expansion of the landfill and environmentally hazardous situations associated with this. Indicates irrational use of resources. As a rule, this method is actively used in underdeveloped and developing countries	Yes	Yes	Demonstrates the level of development of the country quite well
6	Recovery—energy recovery (R1)	Used primarily as fuel or other means of energy production using any available method in accordance with the development of the country	Yes	Yes	Demonstrates the level of development of the country quite well
7	Recycling	According to the values of this indicator, which are presented in Eurostat, this indicator combines Recycling—material and Recycling—composting and digestion	Yes	Yes	Demonstrates the level of development of the country quite well
8	Recycling—material	An effective method of converting any waste into new materials for use and obtaining new products	Yes	No	Included as a component of the Recycling indicator
9	Recycling—composting and digestion	An effective method for managing the organic component of waste with the benefit of restoring the ecological state and soil fertility	Yes	No	Included as a component of the Recycling indicator
10	Preparing for reuse	–	No	No	Lack of data for all countries and for the entire time interval

Consequently, out of 10 waste management operations presented to Eurostat, only four were accepted as diagnostic features, namely: ${X}_{1}$ —Disposal—landfill, ${X}_{2}$—Disposal—incineration (thermal conversion), ${X}_{3}$—Recovery—energy recovery (mainly used as fuel or other means of generating energy), ${X}_{4}$—Recycling.

One of the criteria for selecting diagnostic features is not including variables with low level of variation (the limit of is normally 0.2 or 20%)^32^. In the current study, the variation coefficients for all selected diagnostic features for the time interval studied were more than 0.2 (Table 3).

**Table 3:** Descriptive Statistics.

Descriptive statistics indicators	Diagnostic features	Study year							
		2014	2015	2016	2017	2018	2019	2020	2021
Max	${X}_{1}$	0.813	1.014	0.813	0.845	0.835	0.912	0.826	0.849
	${X}_{2}$	0.105	0.105	0.068	0.038	0.043	0.040	0.026	0.035
	${X}_{3}$	0.524	0.515	0.541	0.529	0.570	0.555	0.601	0.686
	${X}_{4}$	0.660	0.673	0.678	0.671	0.672	0.667	0.704	0.712
Min	${X}_{1}$	0.000	0.000	0.000	0.000	0.000	0.000	0.000	0.000
	${X}_{2}$	0.000	0.000	0.000	0.000	0.000	0.000	0.000	0.000
	${X}_{3}$	0.000	0.000	0.003	0.000	0.000	0.000	0.000	0.000
	${X}_{4}$	0.028	0.000	0.000	0.000	0.000	0.000	0.109	0.113
mean	${X}_{1}$	0.338	0.333	0.307	0.335	0.333	0.331	0.307	0.312
	${X}_{2}$	0.007	0.009	0.008	0.004	0.004	0.004	0.003	0.003
	${X}_{3}$	0.218	0.226	0.249	0.234	0.240	0.240	0.246	0.260
	${X}_{4}$	0.322	0.341	0.370	0.365	0.373	0.381	0.403	0.396
Median	${X}_{1}$	0.321	0.267	0.255	0.307	0.253	0.238	0.229	0.228
	${X}_{2}$	0.000	0.000	0.000	0.000	0.000	0.000	0.000	0.000
	${X}_{3}$	0.196	0.174	0.184	0.190	0.183	0.190	0.208	0.216
	${X}_{4}$	0.306	0.328	0.396	0.375	0.391	0.397	0.409	0.399
Standard deviation	${X}_{1}$	0.277	0.292	0.274	0.296	0.296	0.298	0.286	0.288
	${X}_{2}$	0.020	0.025	0.016	0.010	0.009	0.010	0.006	0.008
	${X}_{3}$	0.182	0.181	0.192	0.185	0.194	0.193	0.198	0.210
	${X}_{4}$	0.162	0.162	0.160	0.163	0.167	0.165	0.159	0.152
Coefficient of variation	${X}_{1}$	0.82	0.88	0.89	0.88	0.89	0.90	0.93	0.92
	${X}_{2}$	2.86	2.78	2.00	2.50	2.25	2.50	2.00	2.67
	${X}_{3}$	0.83	0.80	0.77	0.79	0.81	0.80	0.80	0.81
	${X}_{4}$	0.50	0.48	0.43	0.45	0.45	0.43	0.39	0.38

We assigned weights to selected diagnostic features based on the EU waste management policy and the EU Circular Economy Action Plan^11^, which aims to prevent waste generation and manage it more effectively. According to the EU's "waste management hierarchy," the most desirable scenarios are waste prevention and reuse, followed by recycling (including composting), another recovery (e.g., burning waste to generate energy is a contentious issue in some countries), and disposal, e.g., by landfill, which is the most harmful option for both the environment and health, although one of the cheapest. Thus, the following weights ${\varvec{w}}\left({X}_{j}\right)$ were adopted: ${\varvec{w}}\left({X}_{1}\right)$ = 0.1, ${\varvec{w}}\left({X}_{2}\right)$ = 0.2, ${\varvec{w}}\left({X}_{3}\right)$ = 0.4, ${\varvec{w}}\left({X}_{4}\right)$ = 0.3.

Total amount of generated waste ${{\varvec{y}}}_{{\varvec{i}}}$ per year for each country is equal:

1 $${\varvec{x}}_{{{\varvec{i}}1}} + {\varvec{x}}_{{{\varvec{i}}2}} + {\varvec{x}}_{{{\varvec{i}}3}} + {\varvec{x}}_{{{\varvec{i}}4}} +_{{\varvec{i}}} = {\varvec{y}}_{{\varvec{i}}} ,\;\left( {i = {1}, \, \ldots ,m,j = {1}, \, \ldots ,n} \right)$$

where ${{\varvec{y}}}_{{\varvec{i}}}$—the total amount of generated waste per year for the i-th country; ${{\varvec{x}}}_{{\varvec{i}}{\varvec{j}}}$—waste management operations for the *i*-th country and *j*-th diagnostic feature; ${\upgamma }_{{\varvec{i}}}$—other waste management operations (not included as diagnostic features); *m*—number of countries, varies from 1 to 30; *n*—number of diagnostic features, varies from 1 to 4.

Due to the fact that at different times each country has certain priority waste management operations, and the size of countries and the amount of waste generated in different countries differ significantly, for diagnostic purposes the values of ${{\varvec{x}}}_{{\varvec{i}}{\varvec{j}}}$ were converted to relative values ${{\varvec{a}}}_{{\varvec{i}}{\varvec{j}}}$, where *i* = 1, …, *m*; *j* = 1, …, *n*. So relative values is computed as follows:

2 $${\varvec{a}}_{{{\varvec{ij}}}} = \frac{{{\varvec{x}}_{{{\varvec{ij}}}} }}{{{\varvec{y}}_{{\varvec{i}}} }}$$

The definition of a synthetic indicator (measure) was carried out in several stages^33,34^.

- *Step 1*: The decision matrix ${\left({a}_{ij}\right)}_{m\mathrm{x}n}$ is normalized according to the normalization method
  3 $${\varvec{r}}_{{{\varvec{ij}}}} = \frac{{{\varvec{a}}_{{{\varvec{ij}}}} }}{{\sqrt {\mathop \sum \nolimits_{{{\varvec{i}} = 1}}^{{\varvec{m}}} {\varvec{a}}_{{{\varvec{ij}}}}^{2} } }},(i = {1}, \, \ldots ,m;j = {1}, \, \ldots ,n).$$
- *Step 2*: The weighted normalized decision matrix ${\left({v}_{ij}\right)}_{m\mathrm{x}n}$ is obtained by multiplying normalized matrix with the weights of the attributes:
  4 $${\varvec{v}}_{{{\varvec{ij}}}} = {\varvec{w}}\left( {X_{j} } \right)\cdot{\varvec{r}}_{{{\varvec{ij}}}} ,(i = {1}, \, \ldots ,m;j = {1}, \, \ldots ,n).$$
- *Step 3*: The positive ideal solution (PIS) and the negative ideal solution (NIS) are determined:
  5 $$PIS = \left\{ {v_{1}^{ + } ,v_{2}^{ + } ,..,v_{n}^{ + } } \right\}\;where\quad v_{j}^{ + } = \left\{ {\begin{array}{*{20}c} {\mathop {\max }\limits_{i} \left( {v_{ij} } \right),} & {when\; Cj\; is\; a\; benefit\; attribute} \\ {\mathop {\min }\limits_{i} \left( {v_{ij} } \right), } & {when\; Cj\; is\; a\; cost\; attribute} \\ \end{array} } \right.$$
  6 $$NIS = \left\{ {v_{1}^{ - } ,v_{2}^{ - } ,..,v_{n}^{ - } } \right\}\;where\quad v_{j}^{ - } = \left\{ {\begin{array}{*{20}c} {\mathop {\min }\limits_{i} \left( {v_{ij} } \right),} & {when\; Cj\; is\; a\; benefit\; attribute} \\ {\mathop {\max }\limits_{i} \left( {v_{ij} } \right),} & {when\; Cj\; is\; a\; cost\; attribute} \\ \end{array} } \right.$$
  where i = 1, …, m; j = 1, …, n.
- *Step 4*: The calculation of distances between the PIS ( NIS) and alternatives. The distance values can be measured using the Euclidean distance, which is given as:
  7 $$D_{i}^{ + } = \sqrt {\mathop \sum \limits_{j = 1}^{n} \left( {{\varvec{v}}_{{{\varvec{ij}}}} - v_{j}^{ + } } \right)^{2} } ,(i = {1}, \, \ldots ,m)$$
  8 $$D_{i}^{ - } = \sqrt {\mathop \sum \limits_{j = 1}^{n} \left( {{\varvec{v}}_{{{\varvec{ij}}}} - v_{j}^{ - } } \right)^{2} } ,(i = {1}, \, \ldots ,m)$$
- *Step 5*: The determination of the index ${Q}_{i}$:
  9 $$Q_{i} = \frac{{D_{i}^{ - } }}{{\left( {D_{i}^{ + } + D_{i}^{ - } } \right)}},(i = {1}, \, \ldots ,m)$$
  where $${Q}_{i}\in \left[\mathrm{0,1}\right]$$ ∀i = 1, …, *m*.

Finally, the preferred ranks ${{\varvec{R}}}_{{\varvec{i}}}$ can be obtained according to the similarities to the ${{\varvec{Q}}}_{{\varvec{i}}}$ in descending order to choose the best alternatives.

### Rank significance analysis

We tested the hypothesis about the significance of the obtained ranks using the nonparametric test ${\upchi }_{r}^{2}$– Friedman at the level of statistical significance p < 0.01 using Microsoft Excel 2021, since this method is more efficient than analysis of variance in the case of small samples (up to 30 objects in the sample) and nonnormal distributions.

${\upchi }_{r}^{2}$– Friedman’s test was applied to compare the synthetic indicators ${Q}_{i}$ of each country under study from 2014 to 2021 (*c* = 8 > 3) in the same sample of *m* = 30 EU countries.

10 $$\chi_{r}^{2} = \frac{12}{{mc\left( {c + 1} \right)}}\left( {\tilde{R}_{1}^{2} + \tilde{R}_{2}^{2} + \ldots + \tilde{R}_{{\text{c}}}^{2} } \right) - 3m\left( {c + 1} \right)$$

where ${\widetilde{R}}_{k} =\sum_{i=1}^{\mathrm{m}}{\widetilde{R}}_{ik}$ is the sum of individual ranks for each year, *k* = 1, …, *c*; *c* is the number of years; *m* is the number of countries.

The following hypotheses were accepted.

Hypothesis H0 = {between the values of synthetic indicators obtained by the TOPSIS method, which were measured for each EU country in different years from 2014 to 2021, there are only random differences};

Hypothesis H1 = {there are nonrandom differences between the values of synthetic indicators obtained by the TOPSIS method, which were measured for each EU country in different years from 2014 to 2021}.

Hypothesis H0 is accepted if the observed values of ${\upchi }_{r}^{2}$– Friedman’s test are less than the corresponding critical value chosen at a certain level of statistical significance. It means that the differences in indicators are random.

Hypothesis H0 is rejected (hypothesis H1 is accepted) when the observed values of ${\upchi }_{r}^{2}$– Friedman’s test are more significant than the corresponding critical value selected at a certain level of statistical significance. It means that the differences in indicators are nonrandom, which means that we can investigate the existence of a factor that ensures this nonrandomness.

### Attitude of the Polish population toward waste segregation at the source of generation

Separate waste collection refers to activities that contribute to reducing stored waste and can support improving waste management^18^. Therefore, modern open sources of information were analyzed, such as official reports and scientific studies, to understand Poland's population's attitude to waste segregation at the source of their generation.

**Figure 9:**
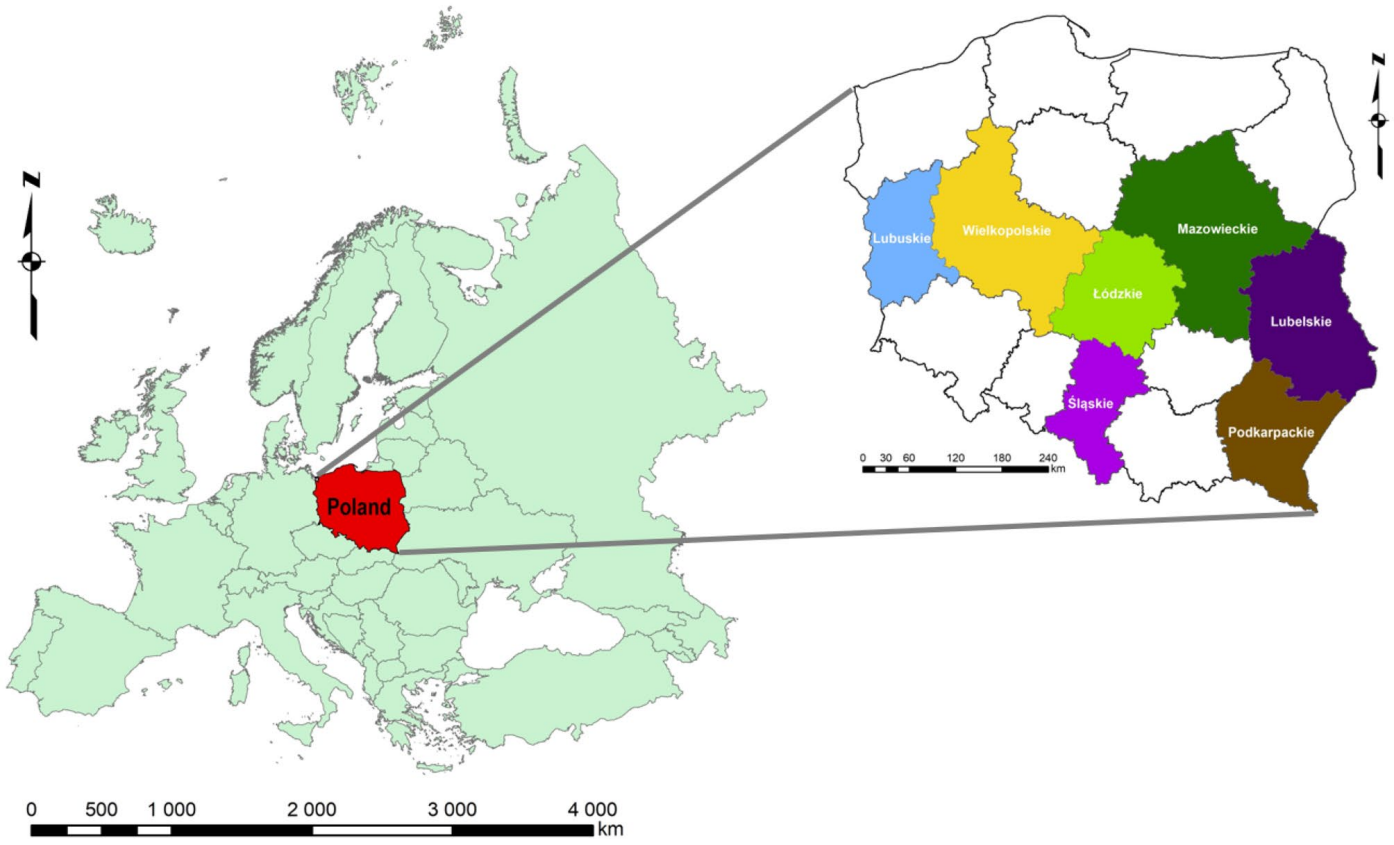
The range of tests (the figure was created by the authors in ArcGIS, background data source: Topographic Objects Database).

All 16 voivodeships in Poland have different levels of waste management. According to the ranking of voivodships in 2015^20^, 4 levels were presented: very high, high, medium, and low. Thus, to analyze the dynamics of selective waste collection by the population in the voivodships of Poland, 2 representative voivodeships from each level were selected following the 2015 study^20^. Since we classified only one voivodeship as very high, 7 were studied (Fig. 9).

### Research limits

The general preliminary analysis of the waste situation in EU countries is limited to 2002–2021, data published by Eurostat. The Central Statistical Office and Eurostat characterize these data as “estimated,” as they may differ from the actual ones due to different moisture content of the waste, as well as with or without taking into account the mass of waste collected from areas without organized waste collection. The ranking of countries by the level of development in waste management was carried out for the time interval from 2014 to 2021, while we did not consider countries such as the Croatia, United Kingdom, Bosnia and Herzegovina, Montenegro, North Macedonia, Albania, Serbia, Kosovo in the ranking due to the lack of data in this time interval. The analysis is based on only four waste management technologies that best demonstrate the country's level in this area (the rationale is presented in the Materials and Methods section).

## Results

### Ranking of EU countries

**Figure 10:**
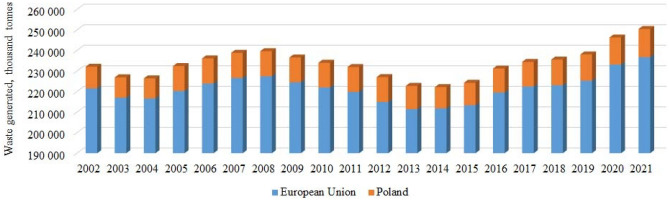
Statistical data on the generation of municipal waste in Poland concerning the total amount in the EU countries for 2002–2021 (data source Eurostat^29^, edited by the authors).

An initial analysis of the dynamics of waste accumulation in Poland over the past 20 years (from 2002 to 2021) shows a periodically changing character (Fig. 10). Clear periods of increased municipal waste production can be observed from 2004 to 2008 and 2014–2021. We observed a similar trend in other EU countries (Table 4).

**Table 4:** Dynamics of changes in municipal waste generation.

Year	EU countries		Poland		Year	EU countries		Poland	
	Thousand tonnes	*Δ, %	Thousand tonnes	*Δ, %		Thousand tonnes	*Δ, %	Thousand tonnes	*Δ, %
2002	221 585		10 509		2012	214 974	− 2.0	12 084	0
2003	217 022	− 2.1	9 925	− 5.6	2013	211 487	− 1.6	11 295	− 6.5
2004	216 689	− 0.2	9 759	− 1.7	2014	211 861	0.2	10 330	− 8.5
2005	220 275	1.7	12 169	24.7	2015	213 409	0.7	10 863	5.2
2006	223 929	1.7	12 235	0.5	2016	219 562	2.9	11 654	7.3
2007	226 623	1.2	12 264	0.2	2017	222 497	1.3	11 969	2.7
2008	227 480	0.4	12 194	− 0.6	2018	223 105	0.3	12 485	4.3
2009	224 543	− 1.3	12 053	− 1.2	2019	225 336	1.0	12 753	2.1
2010	222 009	− 1.1	12 032	− 0.2	2020	233 206	3.5	13 117	2.9
2011	219 839	− 1.0	12 129	0.8	2021	236 801	1.5	13 674	4.2

*Δ is the increase in the amount of waste compared to the previous year.

Reports show a significant increase in the production of waste in Poland by 24.7% in 2005 compared to 2004, then this increase is insignificant, and since 2008 a gradual and stable decline in the production of municipal waste is visible until 2015. In 2015, there was an increase in collected household waste by 5.2% compared to the previous 2014, and the increase continues steadily, including 2021 (Table 4).

**Figure 11:**
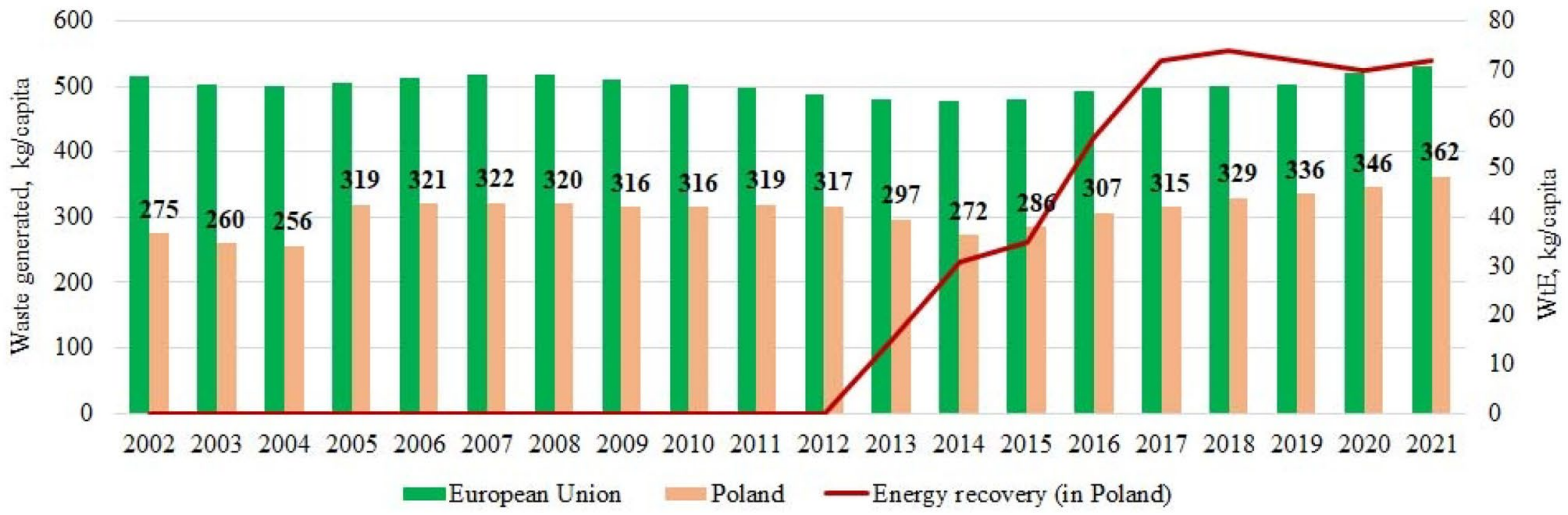
The amount of municipal waste generated per capita for the study period.

The decrease in household waste in 2008, 2009, and 2010 is substantiated by the study^19^ as a decrease in household coverage. However, this may not be the only factor. Research^19^ showed that generated household waste in 2010 accounted for approximately 10% of all generated waste, but not all household waste was collected. The authors also found that in Poland, only a part of the population was covered by household waste collection, which in 2010 was less than 80%. At the same time, we noted that in the same years, the amount of municipal waste per capita in Poland also decreased (Fig. 11), and it is likely that population changes did not significantly affect this trend.

On the other hand, 2008 is known to be the crisis state of the world economy, which also became a financial crisis in the EU countries. According to experts^35^, Poland is the only country in the EU and Central and Eastern Europe (apart from Kosovo and Albania) whose economy was unaffected by this crisis. However, it is possible that the crisis affected some segments of the population and contributed to decreased purchasing power, resulting in a slight reduction in household waste generation. The increase in the waste amount since 2015 can be justified precisely by the fact that for Poland, 2014 was the first full year of the implementation of the amendment to the Law on Maintaining Cleanliness and Order in the Communes, and checks were carried out on the conclusion of contracts with the population for waste collection. That is, the efficiency of the work of bodies in waste management has increased.

EU waste legislation is an essential factor in developing the policies and behavior of the inhabitants of these countries. For example, all interviewed respondents in Austria emphasized the role of regulations in promoting recycling^36^.

At the same time, EU countries have the opportunity to establish their national waste policy, which can be more stringent than EU policy (for example, Austria, Finland, and Sweden, which occupy positions in the top ten countries in waste management)^36^. In addition, the strategic approaches of countries occupying higher positions (Tables 5, 6) are based to a greater extent on the system of segregated waste collection by the population. The fact that waste segregation at the source of their generation is the most effective justifies it^37^. However, as a study^38^ showed, based on an analysis of material and energy flow networks in EU countries, none of the country achieved near-maximum robustness of the material and energy flow networks (that is sustainable circular waste management systems) in the period 2010–2018; and none of the EU countries were recognized as energy self-sufficient in the period from 2004 to 2018 due to. This study notes that only Bulgaria and Germany sometimes had higher resilience values, while the Netherlands has an underlying increased fragility to shocks, despite having a more efficient system for handling its material flows and being a leader in CE in terms of material circularity and energy. Although incineration is not the preferred method because it is not environmentally friendly, in the Netherlands the decision to recycle or incinerate “is one of the most important issues in waste management” and has seen the country significantly reduce landfills and provide district heating and electricity in certain areas. On the other hand, new generation plants for the production of energy from waste are effective for processing large volumes of waste, so the demand for incinerated waste is growing^39^. Thus, in the EU countries there is heterogeneity in waste management, since some countries have settled on waste storage, others on incineration, and still others have a high level of waste recycling and energy recovery from it, although the level of waste generated per capita in these countries is very high.

**Table 5:** The values of the synthetic indicator ${Q}_{i}$ (*i* = 1, …, *m*) for the countries studied (according to the multi-criteria TOPSIS method).

Country	2014	2015	2016	2017	2018	2019	2020	2021
Belgium	0.856	0.843	0.844	0.805	0.514	0.552	0.654	0.570
Bulgaria	0.350	0.363	0.834	0.321	0.285	0.313	0.439	0.338
Czechia	0.394	0.400	0.830	0.420	0.322	0.370	0.345	0.283
Denmark	0.837	0.849	0.822	0.841	0.607	0.647	0.670	0.626
Germany	0.536	0.595	0.822	0.708	0.540	0.584	0.645	0.533
Estonia	0.576	0.700	0.819	0.591	0.459	0.515	0.554	0.471
Ireland	0.665	0.623	0.801	0.622	0.517	0.578	0.576	0.493
Greece	0.322	0.306	0.780	0.227	0.162	0.215	0.185	0.148
Spain	0.446	0.452	0.759	0.394	0.290	0.348	0.368	0.298
France	0.634	0.646	0.702	0.605	0.438	0.483	0.510	0.427
Italy	0.545	0.390	0.680	0.506	0.411	0.460	0.508	0.426
Cyprus	0.323	0.309	0.644	0.214	0.111	0.189	0.169	0.145
Latvia	0.352	0.362	0.609	0.267	0.283	0.326	0.334	0.285
Lithuania	0.404	0.434	0.598	0.509	0.375	0.433	0.516	0.459
Luxembourg	0.628	0.669	0.530	0.824	0.594	0.633	0.635	0.541
Hungary	0.417	0.446	0.501	0.420	0.333	0.379	0.382	0.325
Malta	0.314	0.288	0.437	0.196	0.037	0.038	0.062	0.062
Netherlands	0.864	0.859	0.429	0.815	0.553	0.591	0.652	0.544
Austria	0.769	0.790	0.417	0.787	0.522	0.567	0.631	0.526
Poland	0.337	0.411	0.415	0.467	0.400	0.440	0.483	0.413
Portugal	0.483	0.477	0.406	0.428	0.318	0.372	0.339	0.311
Romania	0.327	0.312	0.355	0.221	0.310	0.355	0.381	0.311
Slovenia	0.394	0.558	0.320	0.448	0.414	0.452	0.572	0.491
Slovakia	0.346	0.349	0.316	0.333	0.323	0.430	0.372	0.325
Finland	0.725	0.778	0.294	0.758	0.579	0.622	0.659	0.591
Sweden	0.853	0.849	0.255	0.840	0.534	0.579	0.627	0.531
Iceland	0.291	0.239	0.252	0.137	0.414	0.399	0.444	0.411
Norway	0.575	0.575	0.250	0.784	0.600	0.640	0.650	0.575
Switzerland	0.895	0.879	0.238	0.856	0.586	0.628	0.648	0.561
Türkiye	0.650	0.706	0.132	0.187	0.209	0.261	0.260	0.208

**Table 6:** Summary table of ranks ${R}_{i}$ according to similarity to ${Q}_{i}$ in descending order to select the best alternatives (*i* = 1, …, *m*) (according to the multi-criteria TOPSIS method).

Country	2014	2015	2016	2017	2018	2019	2020	2021
Belgium	3	5	5	6	11	11	3	4
Bulgaria	23	23	23	23	25	26	19	19
Czechia	20	21	22	20	21	22	24	26
Denmark	5	4	1	2	1	1	1	1
Germany	15	13	12	10	7	7	7	8
Estonia	12	9	11	13	12	12	13	13
Ireland	8	12	14	11	10	9	11	11
Greece	28	28	27	25	28	28	28	28
Spain	17	17	21	21	24	24	23	24
France	10	11	13	12	13	13	15	15
Italy	14	22	19	15	16	14	16	16
Cyprus	27	27	28	27	29	29	29	29
Latvia	22	24	25	24	26	25	26	25
Lithuania	19	19	16	14	18	17	14	14
Luxembourg	11	10	3	4	3	3	8	7
Hungary	18	18	20	19	19	20	20	20
Malta	29	29	29	28	30	30	30	30
Netherlands	2	2	4	5	6	6	4	6
Austria	6	6	9	7	9	10	9	10
Poland	25	20	18	16	17	16	17	17
Portugal	16	16	17	18	22	21	25	22
Romania	26	26	26	26	23	23	21	23
Slovenia	21	15	15	17	15	15	12	12
Slovakia	24	25	24	22	20	18	22	21
Finland	7	7	7	9	5	5	2	2
Sweden	4	3	2	3	8	8	10	9
Iceland	30	30	30	30	14	19	18	18

It should be emphasized that concerning the EU countries, Poland's performance looks more attractive in some years, and according to the ranking results, Poland's position in the ranking among other studied EU countries annually improves from 25th place in 2014 and has been consistently ranked 16th-17th for the last 5 years (Tables 5, 6).

We should note that the quality of municipal waste management is significantly differentiated by country. It is evidenced by the ratio of the maximum synthetic value of ${{\varvec{Q}}}_{{\varvec{i}}}$ to the corresponding minimum at the level for each year under study (Table 7).

**Table 7:** The ratio of the maximum synthetic value to the corresponding minimum.

Parameter	Year							
	2014	2015	2016	2017	2018	2019	2020	2021
Qimax	0.89	0.88	0.84	0.86	0.61	0.65	0.67	0.63
Qimin	0.29	0.24	0.13	0.14	0.04	0.04	0.06	0.06
Qimax/Qimin	3.07	3.68	6.38	6.27	16.28	17.10	10.85	10.18

Thus, we can see that there has been no active development in Poland in recent years in this area, but at the same time, there has been no decrease in this level.

### Testing the hypothesis about the significance of the calculated synthetic indicators

To understand the significance of the ranking results of EU countries obtained during the study period, the ${\upchi }_{r }^{2}$– Friedman’s test was used, which compares three or more matching or paired groups. The results are presented in Table 8.

**Table 8:** The individual ranks of Poland and the sum of the ranks of EU countries as a whole for the period under study according to ${\upchi }_{r }^{2}$– Friedman’s test.

Parameter	Year							
	2014	2015	2016	2017	2018	2019	2020	2021
Individual rank $\widetilde{R}$	8	6	4	2	7	3	1	5
Sum of ranks $\widetilde{R}$	105	91	117	114	202	146	114	191
$\widetilde{R}$ ^2^	11,025	8281	13,689	12,996	40,804	21,316	12,996	36,481

$${\upchi }_{r}^{2}$$(em) (*c* = 8, *m* = 30) = 65.49.

$${\upchi }_{r}^{2}$$(cr) (p < 0,01) = 18.48.

$${\upchi }_{r}^{2}$$(em) > $${\upchi }_{r}^{2}$$(cr).

${\upchi }_{r}^{2}$ – Friedman’s test showed that we should reject the H0 hypothesis about the randomness of synthetic indicators since the calculated value of the test statistics exceeded the critical value at the chosen level of statistical significance *p* = 0.01, i.e., the error of the first kind is less than 1%. Therefore, Poland's level of development in waste management, which it occupies among other EU countries over the studied 8 years, is not accidental and is the result of the influence of certain factors (*p* < 0.01, *m* = 30).

At the same time, ${\upchi }_{r }^{2}$– Friedman’s test allows you to establish that the change in the values of indicators from condition to condition is not random, but it does not indicate the direction of changes and their causes.

### Analysis of the dynamics of selective waste collection by the population in the voivodeships of Poland

Selective waste collection is the basis for more successful and efficient waste management. First, this helps to reduce the number of landfills for waste storage and the number of areas allocated for landfills. In Poland, there is a Decree of the Minister of the Environment No. 1 of December 29, 2016, on a detailed method for the selective collection of individual waste fractions^40^, which specifies which municipal waste is subject to separate mandatory collection; therefore, selective collection of waste by the population would necessarily be carried out to a greater extent or lesser degree.

**Figure 12:**
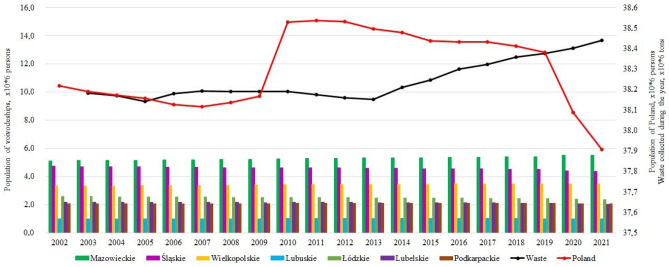
The population of Poland and the total amount of municipal waste for 2002–2021.

According to the Central Statistical Office in Poland (GUS)^30^, which maintains statistics regularly, we can see the following over the past 20 years (2002–2021). The population of Poland (Fig. 11) has had different dynamics since 2002, but after 2010, it has been constantly decreasing, while there has also been a slight decrease in the urban population from 61 to 59%^30^. However, the country's total amount of municipal waste continues to grow (Fig. 12).

**Figure 13:**
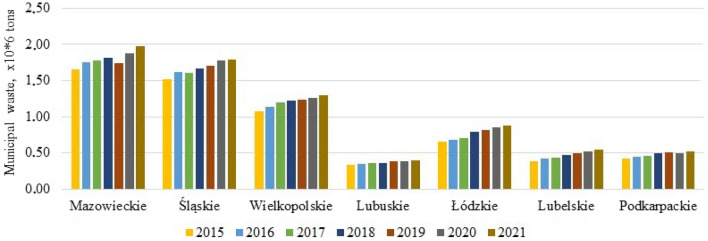
Dynamics of municipal waste in seven voivodships of Poland.

According to the ranking of voivodeships in 2015^20^, the highest level of waste management is Mazowieckie voivodeship. Four voivodships have a high level, among which Śląskie and Wielkopolskie have average indicators, Lubuskie and Łódzkie can act as representatives of the average level of waste management, and Lubelskie and Podkarpackie voivodeships have the lowest level of development. MSW generation is growing in all voivodeships, including the 7 above (Fig. 13).

**Figure 14:**
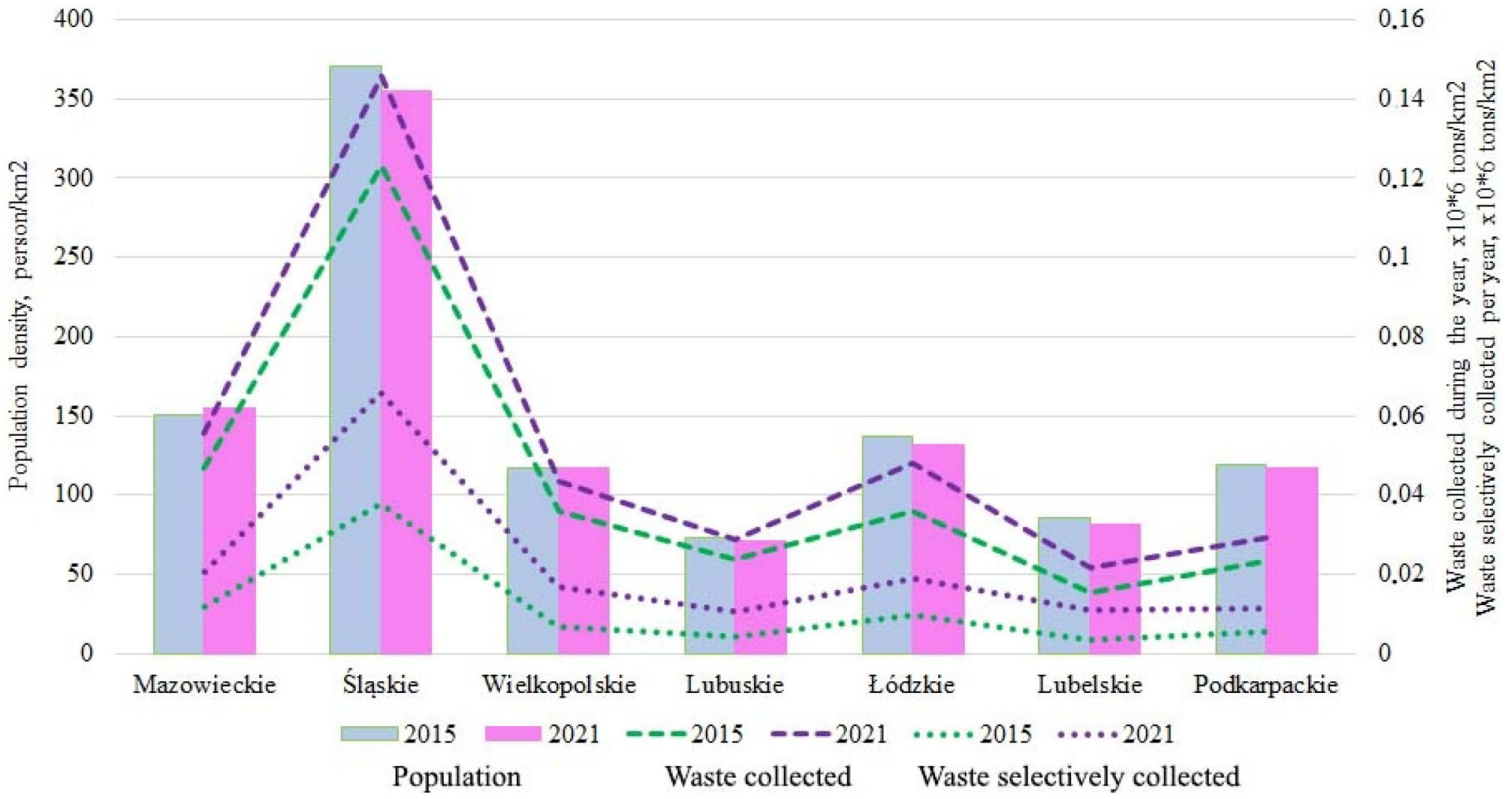
Comparative characteristics of voivodeships.

It is interesting that the population density in these 7 voivodships mainly decreases slightly (by 1.9–4.8%), except for the Mazowieckie (+ 3.1%) and Wielkopolskie (+ 0.7%) voivodships (Fig. 14). We can also note that the total amount of waste per 1 km^2^ and selectively collected waste per 1 km^2^ has increased significantly, with the amount of selectively collected waste per 1 km^2^ increasing by 1.7–3.1 times in 2021 compared to 2015, in contrast to 1.2–1.4 times for the total amount of waste. This proves the positive attitude of residents towards waste segregation at the source. This indicator is reliable because it shows the actual behavior of residents, and not the declarative attitudes that are often obtained in surveys.

Lubelskie became the leader among the studied voivodships, where the total waste production per 1 km^2^ increased by 1.4 times (the decrease in population density is 4.8%), while selective waste per 1 km^2^ increased by 3.1 times, which is 50% of the total waste collected in 2021 compared to 22.7% in 2015 (Table 9). However, no one knows how long the selective collection of municipal waste can be effective without additional incentives and motivation.

**Table 9:** Percentage of selective waste from the total amount of municipal waste generated.

Voivodeship	Selectively collected waste, % of the total	
	2015	2021
Mazowieckie	25.0	37.5
Śląskie	30.7	45.3
Wielkopolskie	18.4	38.9
Lubuskie	18.8	36.3
Łódzkie	27.4	39.4
Lubelskie	22.7	50.0
Podkarpackie	23.5	39.6

Table 9 shows that the amount of mixed waste is decreasing but still significantly prevailing since less than half of the total amount of municipal waste collected was selectively collected.

It is essential that among municipal solid waste, a significant part is food or kitchen waste from households, nonhazardous biodegradable waste from gardens or parks, offices, points and catering establishments or retail stores, as well as comparable waste from the food industry and plants (Table 10).

**Table 10:** The content of biowaste in the total mass of MSW in different countries of the world.

Country	Percentage of organic waste in MSW	References
Nepal	60% food/organic waste 15% paper and cardboard	^1^
Ukraine	45% food/organic waste 15% paper and cardboard 2% wood (garden waste)	^2^
Kazakhstan	11% food/organic waste 7% paper and cardboard	^3^
China	50–70% food/organic waste 9–13% paper and cardboard 1.5–12% wood (garden waste)	^4^
Germany	9.5% food/organic waste 16.8% paper and cardboard 12.7% wood (garden waste)	^41^
Pakistan	56% food/organic waste 2% paper and cardboard	^5^
Türkiye	50.22% food/organic waste 13.3% paper and cardboard	^6^
Argentina	49–57% food/organic waste 3.7–6.3% paper and cardboard 0.2–7.5% wood (garden waste)	^7^
India	53.40% food/organic waste 4.6% paper and cardboard 5.3% wood (garden waste)	^8^
Uzbekistan	53.4% food/organic waste 3.6% paper and cardboard 0.7% Wood (garden waste)	^9^
Poland	17.1–25.3% food/organic waste 12.8–19.1% paper and cardboard 5.3–11.6% wood (garden waste)	^21^ ^42^

## Discussion

In the context of recycling and the circular economy, biowaste can be a source of humus and compost, and most importantly, being highly fermentable, biowaste is a source of energy (in the form of biogas that can be injected into a gas or heating network). Researchers in^43^ predict that by 2024 the total amount of waste will increase by 2%, and the amount of household waste generated will increase by approximately 10%. The amount of waste generated is influenced by the behavior and customs of the residents, including gender, marital status, education and age, education and employment, as well as actions and rules related to waste management^43,44^. Following these predictions and what was said earlier, municipal solid waste can be considered as a sustainable resource for energy production. Thus, in Sweden, it was stated that biowaste processing is a success in waste management; there is also a demand for biogas in the country, which is another important factor^36^. In Finland, the interviewed waste experts stated that biowaste represents the most potential waste stream regarding increased recycling^36^. Therefore, the preferred direction is the segregation collection of such waste, and following Decree^40^, it must be collected selectively.

While other categories of waste include packaging, paper (cardboard), and wood waste, some types of plastics are also of interest as an energy resource^2^ and should be collected selectively.

One of the elements of the selective collection of domestic waste in Poland is a system of points for the collection of oversized, electronic, hazardous, and other municipal waste that does not fall under the categories of "plastic," "metal," "glass," "paper and cardboard," "biowaste".

Household waste selective collection points (HWSCP) operate under the provisions of the Act of September 13, 1996, to maintain cleanliness and order in communities and are designed to receive used items intended for reuse and repair. These facilities, as elements of the waste management system, are funded by the municipal waste management fee of commune's waste management system, although they sometimes charge for replacement items, which may represent additional income to cover operating costs. The HWSCP system in voivodeships is being developed to cover more territories, including rural territories, for the convenience of the population^30^. First, this excludes (prevents) the formation of illegal waste dumps and environmental pollution, and second, it provides a raw material resource ready for processing following its physical and chemical characteristics.

However, a survey of Poles conducted in 2019 showed that 48.4% throw away unnecessary or broken things, 36.5% indicate that they repair things in repair shops, and 31.2% return them to a store or HWSCP^22^. Interestingly, single and semidetached dwellers are likelier to return such items to the store or HWSCP. This way of handling unnecessary or broken items/things in the house is also more common among higher-educated people^22^. Acting as repair points for used items, they are not popular^45^.

At the same time, one should not lose sight of the fact that issues of global importance can be gradually influenced by changing the individual behavior model. Namely, raising public awareness could have a visible effect on waste management. On public awareness issues, many scientific studies have been conducted in different countries, which demonstrate positive dynamics with an increase in public awareness of waste segregation issues^22,36,46^.

For example, in Austria, interviewees emphasized the positive impact of ease of waste collection and the long tradition of selective collection. In Sweden, respondents reported high awareness of citizens about waste recycling and the desire of most citizens to sort their waste^36^.

Regarding the awareness of the population of Poland, then:

- In 2009, 60.2% of respondents reported using reusable bags^47^;
- In 2013, 83% (versus 64% in 2017^48^) of consumers aged 15–74 said they buy precisely as much as they need at the moment^49^;
- In 2014, respondents reported that they preferred not to buy perishable products (54%), more often buy goods produced in the area of residence (52%), use reusable bags (73%), and prefer goods in ecological packaging (50%)^50^;
- In 2019, 33.3% of Poles surveyed said they did not know how to reduce waste, 27.7% considered reducing the amount of waste they produced was not vital to them, and 48.2% avoided disposable/perishable products and declared the use of reusable packages^22^.

Based on the results, we can say that people's behavior is constantly changing depending on random external factors, and at present, there are no traditions among the population in the field of waste reduction. However, a survey^22^ shows that Polish citizens take various actions to minimize the generated waste, and 70.7% provide waste sorting.

## Conclusions

1. The study shows that the production of the total amount of waste in Poland and the amount of waste per capita has constantly been increasing since 2014 while maintaining a stable record of waste and analysis of the situation, providing funding for the operation of the waste management system, and carrying out informative, educational and monitoring work to reduce waste.
2. The conducted research allowed obtaining a positive answer to question 1 posed in the introduction: municipal waste can be one of the sustainable sources of energy in Poland. This is evidenced directly by the increase in energy recovery from waste (R1—Recovery—energy recovery), as well as indirectly by the increase in the amount of waste segregated at source, which allows for methane fermentation of biodegradable waste (R3—Recovery—biological methods), and also increases the efficiency of recovery R1.
3. According to the ranking of EU countries according to the TOPSIS method, it can be seen that in recent years, there has been no active development of Poland in the field of waste management compared to other EU countries, but at the same time, there has been no decrease in this level. Poland has consistently ranked 16th-17th in the last 5 years. At the same time, at the level of statistical significance p < 0.01, there are significant nonrandom differences between the values of synthetic indicators Qi obtained by the TOPSIS method.
4. In each of the 7 surveyed voivodeships in Poland, in the years 2015–2021, there was a significant increase in selective waste collection, and in one of them it was as much as twofold. This proves the positive attitude of residents towards waste segregation at the source of its generation, which indirectly gives a positive answer to question 3, which is one of the research objectives. Raising citizen awareness can visibly affect the selective waste collection, which in turn will make it possible to apply modern recycling methods to them to generate energy, but this needs more research. Although in different years some studies in this area have been carried out, no systematic approach has been found in this direction or systematization of the available data (possibly due to the lack of the required amount of data).
5. It is recommended to carry out studies to determine the sensitivity of the relationship between the actions of the authorities and the generation of alternative energy from waste.

## Data Availability

The study was conducted on the basis of raw statistical data, which is freely available to any researcher who wishes to use them for non-commercial purposes, without violating the privacy of participants, namely: EC data browser website [https://ec.europa.eu/eurostat/databrowser/explore/all/all_themes] and Central Statistical Office (GUS) [https://bdl.stat.gov.pl/bdl/dane/podgrup/themat].
